# Conservation and divergence of the histone code in nucleomorphs

**DOI:** 10.1186/s13062-016-0119-4

**Published:** 2016-04-05

**Authors:** Georgi K. Marinov, Michael Lynch

**Affiliations:** Department of Biology, Indiana University, Bloomington, 47405 IN United States

**Keywords:** Histones, Nucleomorphs, Histone code, Transcription, Chromatin

## Abstract

**Background:**

Nucleomorphs, the remnant nuclei of photosynthetic algae that have become endosymbionts to other eukaryotes, represent a unique example of convergent reductive genome evolution in eukaryotes, having evolved independently on two separate occasions in chlorarachniophytes and cryptophytes. The nucleomorphs of the two groups have evolved in a remarkably convergent manner, with numerous very similar features. Chief among them is the extreme reduction and compaction of nucleomorph genomes, with very small chromosomes and extremely short or even completely absent intergenic spaces. These characteristics pose a number of intriguing questions regarding the mechanisms of transcription and gene regulation in such a crowded genomic context, in particular in terms of the functioning of the histone code, which is common to almost all eukaryotes and plays a central role in chromatin biology.

**Results:**

This study examines the sequences of nucleomorph histone proteins in order to address these issues. Remarkably, all classical transcription- and repression-related components of the histone code seem to be missing from chlorarachniophyte nucleomorphs. Cryptophyte nucleomorph histones are generally more similar to the conventional eukaryotic state; however, they also display significant deviations from the typical histone code. Based on the analysis of specific components of the code, we discuss the state of chromatin and the transcriptional machinery in these nuclei.

**Conclusions:**

The results presented here shed new light on the mechanisms of nucleomorph transcription and gene regulation and provide a foundation for future studies of nucleomorph chromatin and transcriptional biology.

**Electronic supplementary material:**

The online version of this article (doi:10.1186/s13062-016-0119-4) contains supplementary material, which is available to authorized users.

## Background

A common trend in the evolution of endosymbiont genomes is their reduction [1, 2], best exemplified by organellar (chloroplast and mitochondrial) genomes in eukaryotes. This trend is also evident in the fate of secondary endosymbionts (i.e., eukaryotes that have become endosymbionts of other eukaryotes), which have arisen on several occasions in eukaryote evolution [3]. In most lineages bearing secondary plastids, all that remains from the eukaryotic endosymbiont is the plastid, with the nucleus having been lost. There are two notable exceptions to this rule, the chlorarachniophytes and the cryptophytes, which retain a remnant of the endosymbiont’s nucleus and its genome in the form of a nucleomorph [4, 5].

Strikingly, the chlorarachniophyte and the cryptophyte nucleomorphs do not share a common origin, having been acquired from a green and a red alga, respectively, yet their genomes have evolved convergently to a remarkably similar state [6, 7]. Nucleomorph genomes are the smallest eukaryote genomes known, typically just a few hundred kilobases in size; in all known cases, they are organized into three AT-rich chromosomes, with arrays of ribosomal RNA genes in their subtelomeric regions; they are extremely compacted, with almost nonexistent intergenic spaces (sometimes genes actually overlap), and even the genes themselves are frequently shortened [8–14]. These generalizations can be made thanks to the availability of fully sequenced nucleomorph genomes from several species: the chlorarachniophytes *Bigelowiella natans* [13] and *Lotharella oceanica* [12], and the cryptophytes *Guillardia theta* [9], *Hemiselmis andersenii* [14], *Cryptomonas paramecium* [10], and *Chroomonas mesostigmatica* [11].

While much has been learned from the analysis of these genome sequences, numerous important questions about nucleomorph biology remain unresolved. One such issue concerns the mechanisms of transcription and transcriptional regulation in such a crowded and compact genomic environment. All steps of the transcriptional cycle, as well as virtually every aspect of gene regulation in eukaryotes, involve the participation of the histone proteins around which DNA is packaged, usually through their posttranscriptional modifications (PTMs). PTMs are deposited in a regulated manner, most often (but not only) in the unstructured N-terminal tails of histones. They serve as recruitment platforms for effector proteins, which have specific recognition domains for certain marks, thus generating the so called “histone code” [15]. Hundreds of histone modifications have been identified [16] and while most of them are yet to be functionally characterized in detail, the role of a significant number of histone marks is well understood. These include specific marks associated with transcription initiation, transcription elongation, the formation of repressive heterochromatin, mitotic condensation of chromosomes, and other processes [17]. Importantly, most of the posttranscriptionally modified residues are deeply conserved among the majority of eukaryotes [18], implying the ancient origins the histone code and the major functional importance of each individual histone mark.

The extent to which these modifications are conserved in nucleomorphs may provide valuable insights into their biology. Some of the open questions are: 1) whether transcription initiation proceeds through similar mechanisms as in conventional eukaryote nuclei, where the one or two nucleosomes around the transcription start site acquire a specific set of modifications [19] (the tightly packed nucleomorph genes simply do not provide sufficient space for this arrangement given the ∼146 bp minimal footprint of the nucleosome); 2) whether the complex cycle of histone modifications and nucleosome displacement associated with transcription elongation by RNA Polymerase II [20–22] is conserved and operates over the very short nucleomorph genes; 3) whether repressed heterochromatin, which is associated with telomeres, pericentromeric areas, and considerable portions of the rest of the genome in many eukaryotes [23–25] exists in nucleomorphs; 4) whether the mechanisms for regulating chromosome condensation during cell division [26] are conserved in nucleomorphs; 5) whether the patterns of conservation and divergence from the common eukaryotic state are as convergent between chlorarachniophyte and cryptophyte nucleomorphs as are so many other features of their genomes and biology.

This study addresses these questions by analyzing the sequence of nucleomorph histone proteins and the conservation of the key amino acid residues involved in these processes. Chlorarachniophyte nucleomorph histones appear to have lost almost all major histone modifications that are core to the functioning of the histone code. Cryptophyte nucleomorph histones have retained the capacity for a larger number of the key chromatin marks, with the exception of *Cryptomonas paramecium*, whose histone proteins are most divergent within that group. In addition, in all species (both in chlorarachniophytes and in cryptophytes), the heptad repeats in the C-terminal domain (CTD) tail of the largest subunit of RNA Polymerase II, which play a key role in many aspects of the transcriptional cycle and mRNA processing [27, 28], are absent. These results suggest that the transcriptional machinery in nucleomorph exists in a highly derived state relative to its ancestral eukaryotic condition.

## Results

### Histone genes in nucleomorphs

Histone genes were identified in nucleomorph assemblies using a combination of HMMER domain scans [29, 30] and BLASTP [31] searches. It has been previously shown that in *Bigelowiella natans* histones H2A and H2B are encoded in the host nucleus and imported into the nucleomorph [32]. Consistent with these reports and the existing annotations, only histones H3 and H4 can be detected in both chlorarachniophyte nucleomorph genomes. In contrast, all cryptophyte nucleomorphs also encode a version of H2B, implying that only H2A has been transferred to the host nucleus in this lineage. Nucleomorph histones exhibit unique patterns of divergence relative to conventional nuclear histones as evident from their phylogenetic relationships (Additional file 1: Figures S1, S2, S3 and S4). No apparent linker histone genes were found in any of the nucleomorphs.

**Fig. 1:**
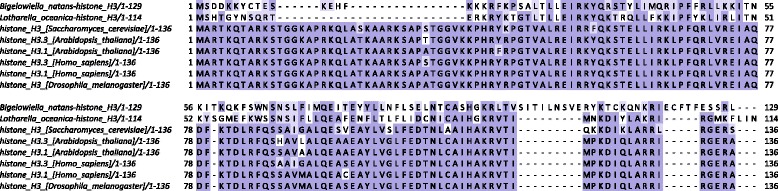
Multiple seqience alignment of chlorarachniophyte nucleomorph histones H3 and nuclear H3 histones from several representative eukaryotes. Multiple sequence alignments were carried out using MUSCLE [102] and visualized using Jalview [103]

**Fig. 2:**
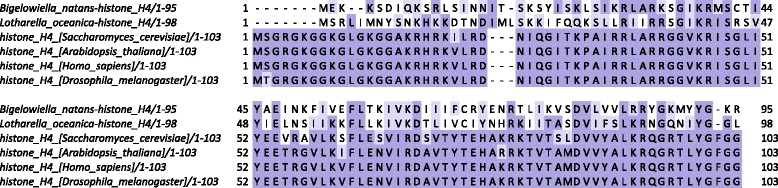
Multiple seqience alignment of chlorarachniophyte nucleomorph histones H4 and nuclear H4 histones from several representative eukaryotes. Multiple sequence alignments were carried out using MUSCLE [102] and visualized using Jalview [103]

To gain insight into their functional properties, multiple sequence alignments of nucleomorph histones were carried out, using nuclear core histones from several other eukaryotes, in which the role of histone marks in chromatin biology has been extensively studied, as reference. Figures 1 and 2 show these alignments for chlorarachniophyte histones H3 and H4. In both species, a significant portion of the N-terminal tail of histone H3 (between residues 10 and 38) is missing, and overall the sequence is highly divergent. Surprisingly, the *Bigelowiella* histone H3 has a 10-amino acid insertion in the core histone part of the protein, which is not shared with *Lotharella*; its significance is not clear at present. The properties of H4 histones are similar: both species share a ∼10-amino acid deletion at the very end of the N-terminal tail and the sequences are highly divergent from those of other eukaryotes.

**Fig. 3:**
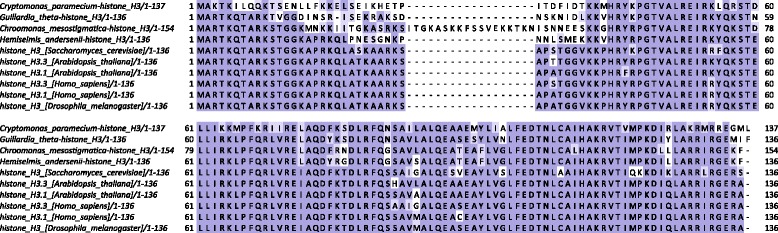
Multiple seqience alignment of cryptophyte nucleomorph histones H3 and nuclear H3 histones from several representative eukaryotes. Multiple sequence alignments were carried out using MUSCLE [102] and visualized using Jalview [103]

Cryptophyte histone H3 sequences are generally much more similar to conventional eukaryotic histone H3 than those of chlorarachniophytes, yet they also exhibit numerous unconventional features (Fig. 3). The extreme N-terminal end is particularly well conserved, with the exception of that of *Cryptomonas*’s H3, yet all species display significant sequence divergence roughly coinciding with the deleted region in chlorarachniophyte H3 proteins, including a large insertion in *Chroomonas*.

**Fig. 4:**
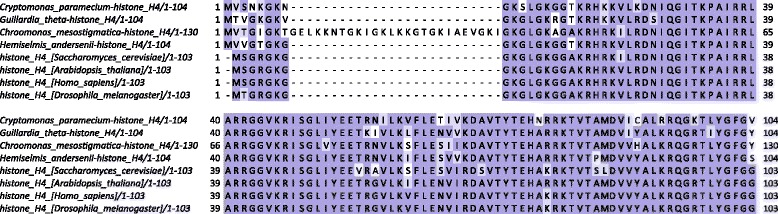
Multiple seqience alignment of cryptophyte nucleomorph histones H4 and nuclear H4 histones from several representative eukaryotes. Multiple sequence alignments were carried out using MUSCLE [102] and visualized using Jalview [103]

*Chroomonas* also has large insertions in the N-terminal tail of its histone H4 (Fig. 4) though the rest of the sequence is relatively similar to that of other eukaryotes, as are those of the other three cryptophytes.

Unlike chlorarachniophytes, cryptophytes also have a nucleomorph-encoded histone H2B protein. It should be noted that higher levels of sequence divergence are observed for H2A and H2B proteins within eukaryotes in general. Nevertheless, their conservation characteristics in cryptophytes are similar to those of cryptophyte H3 and H4 nucleomorph histones (Additional file 1: Figure S5), with significant divergence seen in the N-terminal tail (which is completely missing in *Cryptomonas* and *Guillardia*, and has large deletions in *Chroomonas* and *Hemiselmis*).

### The histone code in nucleomorphs

**Fig. 5:**
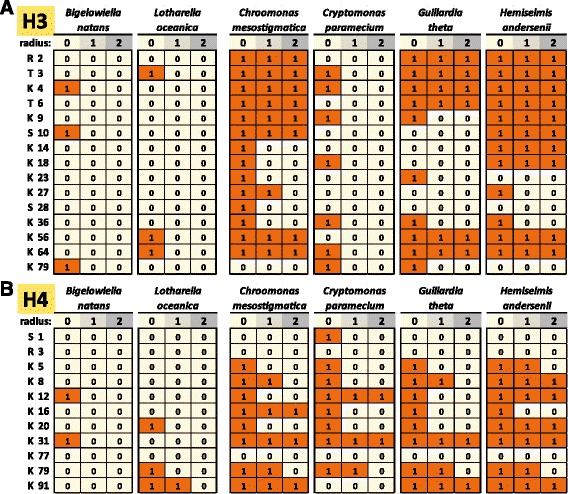
Conservation of key posttranscriptionally modified residues in chlorarachniophyte and cryptophyte nucleomorph histones H3 and H4. **a** Histone H3; **b** Histone H4. The radius *r* refers to the size of the context considered when scoring conservation. When *r*=1, only the residue itself is considered; when *r*=0, a perfect match to the three-amino acid peptide also including the flanking residues on each side is required; when *r*=2, the five-amino acid peptide also including the two flanking residues on each side is considered. A score of 1 means conservation according to these criteria, while a score of 0 means lack of conservation. Conservation was scored against the *Homo sapiens* histone H3.3 and H4 sequences

To assess the conservation of the histone code in nucleomorphs, the preservation of modified residues and their immediate three- and five-amino acid sequence contexts was analyzed. There are some limitations to this approach. While the conservation of a given residue does not necessarily mean that its post-translational modifications are also conserved, its absence does mean that the corresponding modifications have been lost. In addition, sequence context conservation can be due to the presence of independent constraints on the residue and its neighborhood rather than because of conservation of its posttranscriptional modifications. Nevertheless when observed, such conservation makes it more plausible that the capacity for depositing the modification has been retained. The conservation of the best functionally understood residues on histones H3 and H4 in chlorarachniophyte and cryptophyte nucleomorphs as evaluated according to these criteria is shown in Fig. 5.

#### Histone marks associated with heterochromatin

In most eukaryotes, heterochromatin occupies the pericentromeric and telomeric regions of chromosomes and is also used as a mechanism for silencing transposable elements as well as some host genes. Histone marks play a central role in the formation of heterochromatin, in particular the trimethylation of histone 3 lysine 9 (H3K9me3), which classically serves as a recruitment platform for the HP1 heterochromatin protein [33–35]. Other marks associated with heterochromatin include H4K20me3 [35, 36] and H3K64me3 [37]. In addition, another repressive mechanism operates through the H3K27me3 mark, which is key to Polycomb complex-mediated gene repression in both plants and metazoans [38, 39]. It should be noted that different combinations of marks might be associated with distinct types of heterochromatin in multicellular eukaryotes [40, 41].

Strikingly, H3K9 and H3K27 are completely absent in chlorarachniophyte nucleomorphs. H4K20 and H3K64 are observed in *Lotharella*, but their sequence context is not conserved.

In cryptophytes, H3K9 and its sequence context are conserved in *Chroomonas* and *Hemiselmis*; H3K9 is present in *Cryptomonas* and *Guillardia*, but the sequence context is not conserved. H3K27 is absent from *Cryptomonas* and *Guillardia*, and present in *Chroomonas* and *Hemiselmis*, but without the context being conserved. H3K64 is well conserved in cryptophytes with the exception of *Cryptomonas*, while H4K20 is present in all species but its context is perfectly conserved only in *Hemiselmis*.

#### Phosphorylation marks associated with chromosome dynamics during mitosis

Histone proteins contain multiple phosphorylation sites, which play an important role in the regulation of the dynamics of chromosome condensation during mitosis. These include H3T3ph [42–44], H3S10ph [45–48], H4S1ph [49], and others [50–53]. In addition, H3S28ph is associated with transcriptionally active chromatin [54, 55].

These marks seem to be largely absent from chlorarachniophyte histones (H3T3 is present in *Lotharella* and H3S10 in *Bigelowiella*, but no sequence context conservation is observed; H4S1 is absent from both species).

*Chroomonas*, *Guillardia* and *Hemiselmis* retain H3T3, H3S10 is conserved in *Chroomonas* and *Hemiselmis* but absent from the more divergent tails of *Cryptomonas* and *Guillardia*. H4S1 is also absent from cryptophytes.

H3S28 is nearly absent from all nucleomorphs.

#### Histone marks associated with the transcription elongation cycle

The process of transcription faces a major barrier in eukaryotes in the form of the nucleosome, which inhibits transcriptional elongation. A complex cascade of molecular events, which involves a number of histone modifications, has evolved in response to this constraint. Acetylation marks are deposited on nucleosomes in front of the elongating polymerase, in order to facilitate the opening of chromatin. The FACT complex [56, 57] plays the role of a histone chaperone, partially disassembling nucleosomes so that the polymerase can transcribe through them, then reassembling them. This process also involves the monoubiquitination of histone H2B at a specific site (H2BK120ub1 [58, 59]). Transcriptional elongation is also associated with the deposition of H3K36me3 [60, 61], which plays an important role in recruiting histone deacetylases after the polymerase has passed [62–64] so that the newly deposited acetylation marks can be removed and potential cryptic transcription repressed [65]. Methylation of H3K79 is also associated with transcriptional elongation [66–69]. Finally, in addition to its role in elongation, H3K36me3 seems to also be involved in the regulation of splicing and RNA processing [70–75].

Remarkably, H3K36 is completely absent in chlorarachniophytes, and is present, but with a poorly conserved sequence context, in cryptophytes. H3K79 is observed in *Bigelowiella*, *Cryptomonas*, and *Guillardia*, and its sequence context is moderately conserved, but it is absent from all other species. H2BK120 is present in all cryptophytes (Additional file 1: Figure S6), but without any conservation of the sequence context.

#### Histone marks associated with promoters

The classical marker of eukaryotic promoters is the deposition of H3K4me3 [76, 77]. A lysine residue is present at that position in *Bigelowiella*, but its sequence context is very different, and it has been replaced with a glycine in *Lotharella*.

The three cryptophytes with relatively well-conserved N-terminal tails do possess a correspondingly well-conserved H3K4, the exception being the highly divergent *Cryptomonas* tail.

#### Histone marks associated with activation/repression of transcription

A number of histone marks have been described as having a positive or negative correlation with transcription, either by regulating the modification of other residues (what is often referred to as histone mark “crosstalk”), by generally serving to create a more open chromatin state (acetylation being the primary example), or through other less well understood means. These include methylation on several arginines (H3R2me2 [78–80], H4R3me2 [81, 82]), which can have positive or negative effects on transcription depending on whether the dimethylation is symmetric or asymmetric, H3T6ph [83] and acetylation marks with generally activating effects (H3K14ac, H3K18ac, H3K23ac, H4K5ac, H4K8ac, H4K12ac, H4K16ac, H4K31ac, H2BK5ac, H2BK12ac, H2BK15ac, H2BK20ac, and others), as well as acetylation marks with better understood specific roles (for example, H3K56ac [84–86] and H4K91ac [87], which might also play a role in nucleosome assembly in addition to gene regulation).

All of these residues are either completely missing or poorly conserved in chlorarachniophytes, but display higher levels of conservation in cryptophytes (with the *Cryptomonas* being the exception).

### Absence of RNA Polymerase II CTD heptad repeats in nucleomorphs

The histone code is not the only code playing a major role in transcriptional elongation. It operates in concert with the so called CTD code [88], consisting of the posttranscriptional modifications of the C-terminal domain tail of the largest subunit of RNA Polymerase II (Rpb1). Ancestrally to all extant eukaryotes [89], the CTD domain contains a large number of repeats of the consensus heptad sequence YSPTSPS [90] (note that the repeats are not always perfect matches to the consensus). The heptad contains five phosphorylation sites and two proline isomerization sites. Specific modifications are deposited on the tail during each phase of the transcriptional cycle, and serve to recruit various effector and regulatory proteins [27, 28]. As a result the CTD repeats play a pivotal role in almost all aspects of transcriptional elongation and mRNA processing, and their status in nucleomorphs is of significant interest.

All six nucleomorphs encode an Rpb1 protein. However, all of these proteins completely lack heptad repeats (Additional file 1: Figure S7) indicating a corresponding absence of conservation of the CTD code.

## Discussion

The analysis of nucleomorph histone protein sequences presented here reveals a number of puzzling and potentially fascinating aspects of nucleomorph chromatin biology.

The first major observation is that while nucleomorph histones are generally quite derived in all species, this is much more so in chlorarachniophytes than in cryptophytes, with most of the classic sites of histone modification completely missing in the former, and significantly higher conservation observed in the latter. However, even within cryptophytes one species, *Cryptomonas paramecium*, also exhibits a significant divergence from the conventional state and the loss of most key histone mark residues. The evolutionary forces and biochemical constraints responsible for these patterns remain to be elucidated by future studies.

Second, it seems that typical heterochromatin is most likely absent from nucleomorphs given that the key residues participating in its formation have been lost. This is not entirely surprising – the extremely small size, compact nature and high transcriptional activity [91] of nucleomorph chromosomes presumably eliminates the need for heterochromatin, even in telomeric regions.

A similar relaxation of biochemical constraints might be behind the loss of the phosphorylation sites involved in chromosome condensation during mitosis. The mechanisms of nucleomorph division, its regulation, as well as the chromosome dynamics during the process, remain very poorly understood, so future studies will have to bring more light onto this subject. Still, it is conceivable that nucleomorph chromatin is not compacted in a fashion similar to that of autonomous eukaryote nuclei during mitosis.

The most striking loss of functionality in nucleomorphs is associated with transcription initiation and elongation. The potential for initiation-associated methylation of H3K4 at least exists in cryptophytes, but is certainly lost in chlorarachniophytes. The mechanisms for promoter definition and the details of transcription initiation in this context are at present a mystery. The situation is even more puzzling when it comes to transcriptional elongation. It is possible that H3K79 methylation is happening in cryptophytes, and H3K36 methylation and H2BK120 ubiquitination cannot be confidently ruled out, but they are almost certainly completely absent in chlorarachniophytes. Equally striking is the absence of heptad repeats from the tail of the RNA Polymerase II largest subunit. In total, these observations suggest that both the nucleosome dynamics during transcriptional elongation and the coupling of transcription with mRNA processing have evolved to a highly derived state in nucleomorphs. One possibility is that the high transcriptional activity of nucleomorphs allows for a permanently open nucleosome state; however, it is not clear how cryptic transcription can be prevented if that is the case, and such a hypothesis only explains the absence of H3K36me3, but not that of Rpb1’s CTD repeats, which are crucial for a wide variety of other processes in addition to the acetylation/deacetylation cycle during elongation.

The poor conservation of many of the residues known primarily for being acetylated (and not yet linked to specific functions beyond that) is also not entirely surprising, as the role of opening chromatin can probably be taken over by other lysine residues (indeed, studies in yeast in which lysine residues have been systematically mutated have shown substantial redundancy between acetylation sites [92, 93]). Histone acetylation is almost certainly happening in nucleomorphs, as all six nucleomorph genomes encode a single histone deacetylase protein, histone acetyltransferases are detected in all cryptophyte nucleomorphs (Additionalf file 1: Figure S8), and additional ones might be imported from the host (although it is possible that they are retained because of their importance for the acetylation of non-histone proteins and not of histones themselves). Unfortunately, the problem of identifying the complete set of readers and writers of epigenetic marks in nucleomorphs is currently quite difficult because it is not known how many and which proteins are imported from the host. Thus while the presence of a given protein in the nucleomorph genome confirms its presence in the nucleomorph, its absence from the genome does not necessarily mean that it is not imported from the host; the histone genes themselves are a prime example of the ubiquity of this phenomenon.

Histone mark readers, writers, and erasers include the Dot1 methyltransferase, which methylates H3K79, no examples of which are found in nucleomorphs genomes (Additional file 1: Figure S8); SET domain proteins, which deposit all other lysine methylations [94], two of which are found in *Chroomonas*, *Guillardia*, and *Hemiselmis* nucleomorphs, but none in *Cryptomonas paramecium* and the chlorarachniophytes; histone acetyltransferases (HATs) [95] (found in cryptophyte nucleomorph genomes but not in chlorarachniophytes); the sirtuin deacetylases [96] (also not present in any of the species); bromodomain-containing proteins, which read acetylation marks [97] (found in some cryptophyte nucleomorphs); chromodomain-containing proteins, which read lysine methylated residues [98] (none found in nucleomorphs); PHD finger proteins, readers of lysine and arginine methylations [99] (also not present), and the Jumonji and LSD1 families of lysine demethylases [100] (not present in nucleomorphs either). A putative SWI/SNF chromatin remodeling protein is identified in most nucleomorphs, as is a Hira histone chaperone [101] in both cryptophytes and *Bigelowiella* as well as an ASF1 histone chaperone [101] in cryptophytes (Additional file 1: Figure S8). No FACT homologs are identified, and neither are DNA methyltransferases or other typical eukaryotic DNA methylation-related proteins (methyl-binding domains), which read DNA methylation in eukaryotes.

## Conclusions

Overall, the observations presented here suggest that a substantial fraction of the epigenomic toolkit has been lost from nucleomorphs (consistent with the divergence of their histone sequences) and much of whatever remains of it has been transferred to the nucleus, as has happened to many other genes previously resident in the nucleomorph genome. The loss of the code has most likely happened in concert with a loss of at least some of the chromatin remodeling and regulation functionality that it is normally a part of. However, how much exactly has been lost remains to be determined by future experiments. Given that some of the processes concerned are fundamental to the biology of transcription in eukaryotes, it is not easily envisioned how they could have been lost. One tantalizing possibility is that even if much of the conventional histone code has been lost, substantial innovation has occurred in parallel, and other residues have assumed the roles previously played by the classical modified residues known from autonomous nuclei. The several large insertions in histone proteins observed in *Chroomonas*, containing a number of lysine residues, lending some support to such an idea.

Future studies utilizing the power of modern proteomics and functional genomics should provide insight into these questions, by identifying the post-translational modifications of nucleomorph histones, and by mapping the chromatin structure, the distribution of modified histones, and the positions and identities of regulatory and transcriptional elements in nucleomorph genomes.

## Methods

Except where otherwise stated, all analyses were performed using custom-written python scripts.

Nucleomorph genome sequences were obtained from the NCBI nucleotide database (GenBank: DQ158858.1, GenBank: DQ158857.1, GenBank: DQ158856.1, GenBank: CP003682.1, GenBank: CP003681.1, GenBank: CP003680.1, GenBank: CP002174.1, GenBank: CP002173.1, GenBank: CP002172.1, GenBank: NC_002753.1, GenBank: NC_002752.1, GenBank: NC_002751.1, GenBank: CP000883.1, GenBank: CP000881.1, GenBank: CP000881.1, GenBank: CP006629.1, GenBank: CP006628.1, GenBank: CP006627.1).

Histone genes were annotated using a combination of HMMER3.0 [29] scans against the Pfam 27.0 database [30] (scanning for histone fold domains) and BLASTP [31] searches (using histone sequences from *Homo sapiens*, *Saccharomyces cerevisiae*, *Drosophila melanogaster*, and *Arabidopsis thaliana* as queries). Multiple sequence alignments were carried out using MUSCLE [102] and visualized using Jalview [103].

Chromatin modification and remodeling proteins were identified using HMMER3.0, with an e-value cutoff of 10^−8^, as well as an additional BLAST search using chromatin proteins from *Homo sapiens*, the red alga *Cyanidioschyzon merolae* and the green algae *Chlamydomonas reinhardtii* and *Micromonas pusilla* as queries.

## Reviewers’ comments

### Lakshminarayan Iyer (Reviewer 1)

Marinov and Lynch provide a useful analysis of the nucleomorph histone code using the existing completely sequenced nucleomorph genomes. By comparing histone sequences, the study brings into focus the unique aspects of transcriptional/chromatin regulation in these nucleomorphs. In particular, one can posit what type of modifications are likely to be retained in these reduced genomes, of which some might be provided from their eukaryotic hosts. The sequence analysis of the histones is thorough. I do have a few additional pointers that pertains to the discussion and supplementary Figure 4. Comments: 1. The list of domains involved in post-translational modifications that were analyzed isnt complete (For other domains including readers and modifiers see PMIDs: 21507350, 21507349, 19092802). It would be prudent to check domains such as CXXC, LSD1, SAD/SRA and so on. 2. A word of caution: some PTM proteins and readers can diverge, so searches with individual proteins as starting points in addition to running automatic profiles should be done. For example, I detected a GCN5 acetyltransferase in the Guillardia theta nucleomorph (gi: 162606188) possessing both the acetylase and bromo domains. This is in line with the conservation of lysine residues in H3 such as H3K9, H3K14 and H3K23 that are substrates for this acetylase, in this nucleomorph. I have not checked the other nucleomorph genomes, but it might be prudent to check individual genomes carefully for divergent homologs.

Authors response: *Thank you for you suggestions. The analysis of chromatin modifying and remodeling proteins has been updated accordingly.*

### Berend Snel (Reviewer 2)

#### Reviewer summary

The authors present an in depth analysis of the alignments of the histone proteins of nucleomorph genomes to reveal the evolutionary fate of the histone code of these genomes. Although the histone residues provide interesting and relevant discussion, I missed certain analyses and information which I think should be added. Moreover the results are somewhat limited as it basically consists of 2 alignments with some textual description of histone modifying enzymes, and without additional phylogenetic analysis.

#### Reviewer recommendations to authors

Upon first reading the manuscript quite some relevant information which at least for me was necessary to understand the story was missing or only presented in the discussion. Specifically the following issues are I think unclear or missing but needed for context or as results. First that the machinery for depositing relevant histone marks is needed to modify the histones, and to what extent this machinery is present on the nucleomoprh genome or the host nuclear genome. And if it is present on the nuclear genome, to what extend these proteins might or might not be targeted to the nucleomoprh nucleus. These points are to a certain extent present in the discussion, but part of these points are results and should be in the results (e.g. the histone modification enzymes encoded by the nuclear host genome as discussed on page 8). Other points are not sufficiently discussed at all while they are relevant for the interpretation. I specifically think that the manuscript insufficiently discusses what we know about proteins being translocated to the nucleomorph. What signals are present in sequences being targeted to the nuceomorph and if bioinformatics or proteomics can or has helped in identifying such proteins.

Authors response: *The reasons we did not do a thorough analysis of the nuclear genomes are that first, we only have nuclear genome sequences for one chlorarachniophyte (Bigelowiella) and one cryptophyte species (Guillardia), thus there was no way to generalize results over the whole of each group, and second and more important, in silico nucleomorph targeting predictions are very far from being reliable. Direct proteomic measurements exist only for the Bigelowiella natans nucleomorph [*104*] and that particular study only identified 324 proteins. Of those, only half had predicted SP and/or TP targeting sequences, and the imported histones and transcription factors had no obvious such sequences at all.*

After reading the manuscript I was convinced that the nucleomorph histones are indeed quite diverged but this information cannot be fully interpreted if its (predicted) proteomic context is not at least partially provided. Related to the previous point. Obviously the nuclear host genome can also encode proteins of nucleomorph origin. And some of these might still be active in the nucleomorph cytoplasm or nucleus. In addition, the nucleomorph encoded histones of especially the chlorarachniophyte are so diverged I was wondering if they are in fact even orthologous to H3 or H4. Both of these issues could perhaps be clarified by constructing gene trees (although that is exceedingly difficult for histone proteins and especially for such highly diverged proteins). Nevertheless it would be very interesting if such a gene tree would show for example a host nuclear encoded protein in Bigelowiella that is closely related to the green algae. Even in the absence of any firm biochemical knowlegde on the transfer mechanism such a gene would provide good candidates for a potential histone protein that could be active in the nucleomorph nucleus and could “rescue” the diverged nucleomorph encoded protein. A quick panther scan of the Bigelowiella genome with the H3 profile finds 9 predicted histone H3 proteins. This number indeed raises the question of paralogy, orthology and orther “pseudo” orthologs originated by nuclear transfers of secondary endosymbiosis.

Authors response: *We did not initially carry out a phylogenetic analysis of all Bigelowiella and Guillardia histone proteins because that has been previously done in Hirakawa et al. 2011, while our focus was on the functional inferences we can generate based on the sequences, as a starting point towards a future experimental dissection of that functionality. An updated such analysis will be of great interest in the future when more nuclear genomes become available, but at the moment there are only these two. We have included phylogenetic trees of the relevant sequences using transcriptome assemblies from the Marine Microbial Eukaryote Transcriptome Sequencing Project (MMETSP) [*105*] as supplementary figures in the revised manuscript.*

#### Minor issues

I do not understand why the alignments in Figs. 1, 2 and 3 are presented in the way they are. i.e. why is the for example the alignment of reference eukaryote H3 with histone H3 of Bigelowiella a separate alignment from that of Lotharella? I see no reason why this could not be a single alignment. Normally I would also expect the cryptophyte histones to be in a single alignment with the reference species.

Authors response: *The figures have been modified according to the reviewer’s suggestions.*

## Additional file

Additional file 1 Supplementary Figures. (PDF 7127 kb)

## Abbreviations

A: adenine
CTD: C-terminal domain
HAT: histone acetyltransferase
MMETSP: Marine Microbial Eukaryote Transcriptome Sequencing Project
PTM: posttranscriptional modification
RNA: deoxyribonucleic acid
RNA: ribonucleic acid
SP: signal sequence
T: thymine
TP: transit peptide sequence
