# Supplementary material for: Conservation and divergence of the histone code in nucleomorphs
Source: Biol Direct. 2016 Apr 5;11:18. doi: 10.1186/s13062-016-0119-4 (PMC4822330; doi:10.1186/s13062-016-0119-4)
Supplement: Additional file 1 — Supplementary Figures. (PDF 7127 kb) [file 13062_2016_119_MOESM1_ESM.pdf]

Supplementary Figures

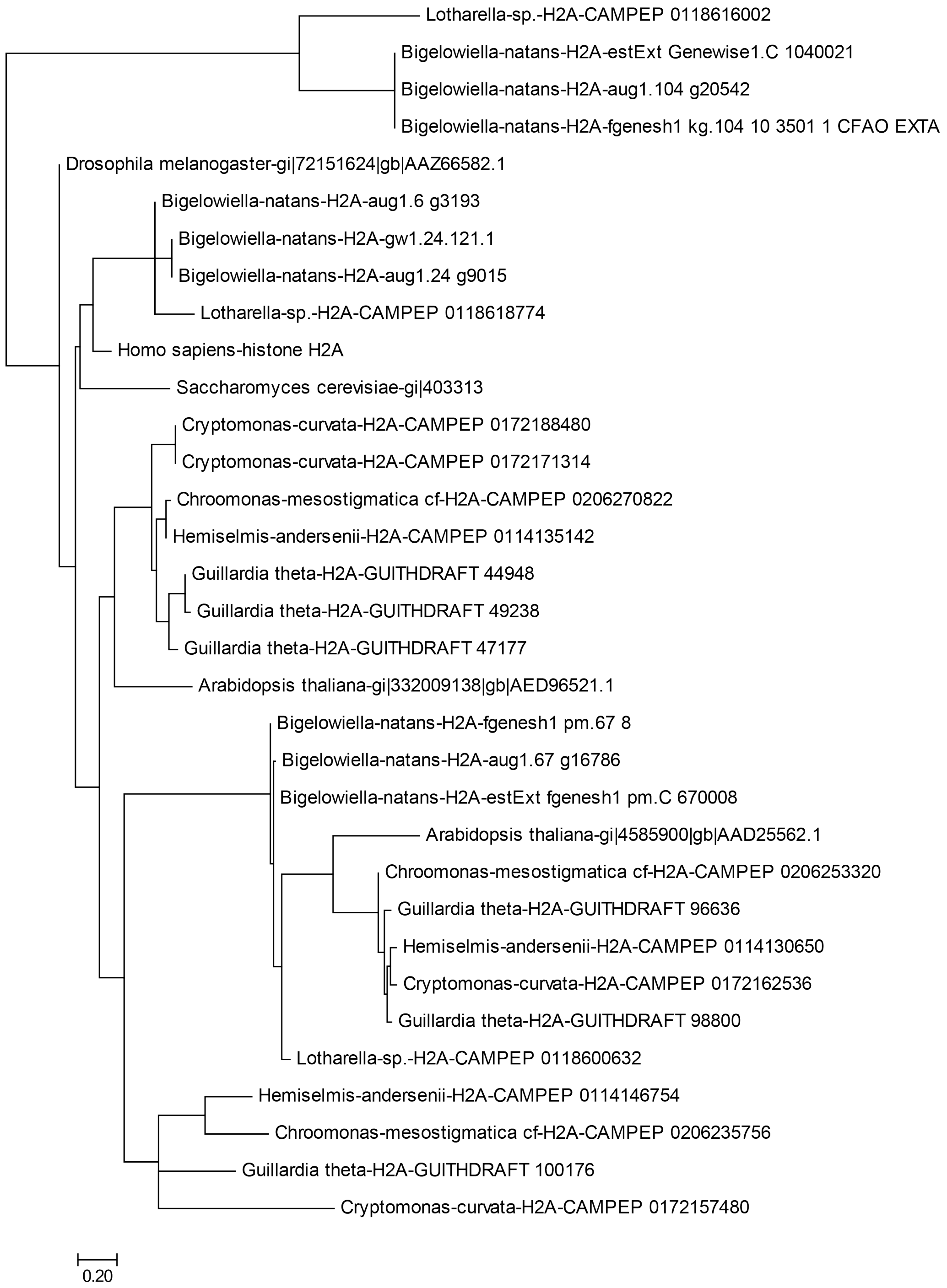

Supplementary Figure 1: Maximum likelihood phylogenetic tree of all nucleus-coded H2A protein variants in cryptophytes and chlorarachniophytes. The tree was generated using MUSCLE (Edgar 2004) multiple sequence alignments and RAxML (Stamatakis 2014; version 8.0.26) under the LG+G model and with 100 bootstrap replicates, and visualized using MEGA (Tamura et al. 2013). H2A sequences were obtained from available genome assemblies for *Bigelowiella natans* and *Guillardia theta* and from transcriptome assemblies for *Lotharella* sp., *Hemiselmis andersenii*, *Cryptomonas paramecium*, and *Chroomonas mesostigmatica* provided by the Marine Microbial Eukaryote Transcriptome Sequencing Project (MMETSP) (Keeling et al. 2014) (after filtering out incompletely assembled proteins). Canonical H2A sequences from several other eukaryotes were also included.

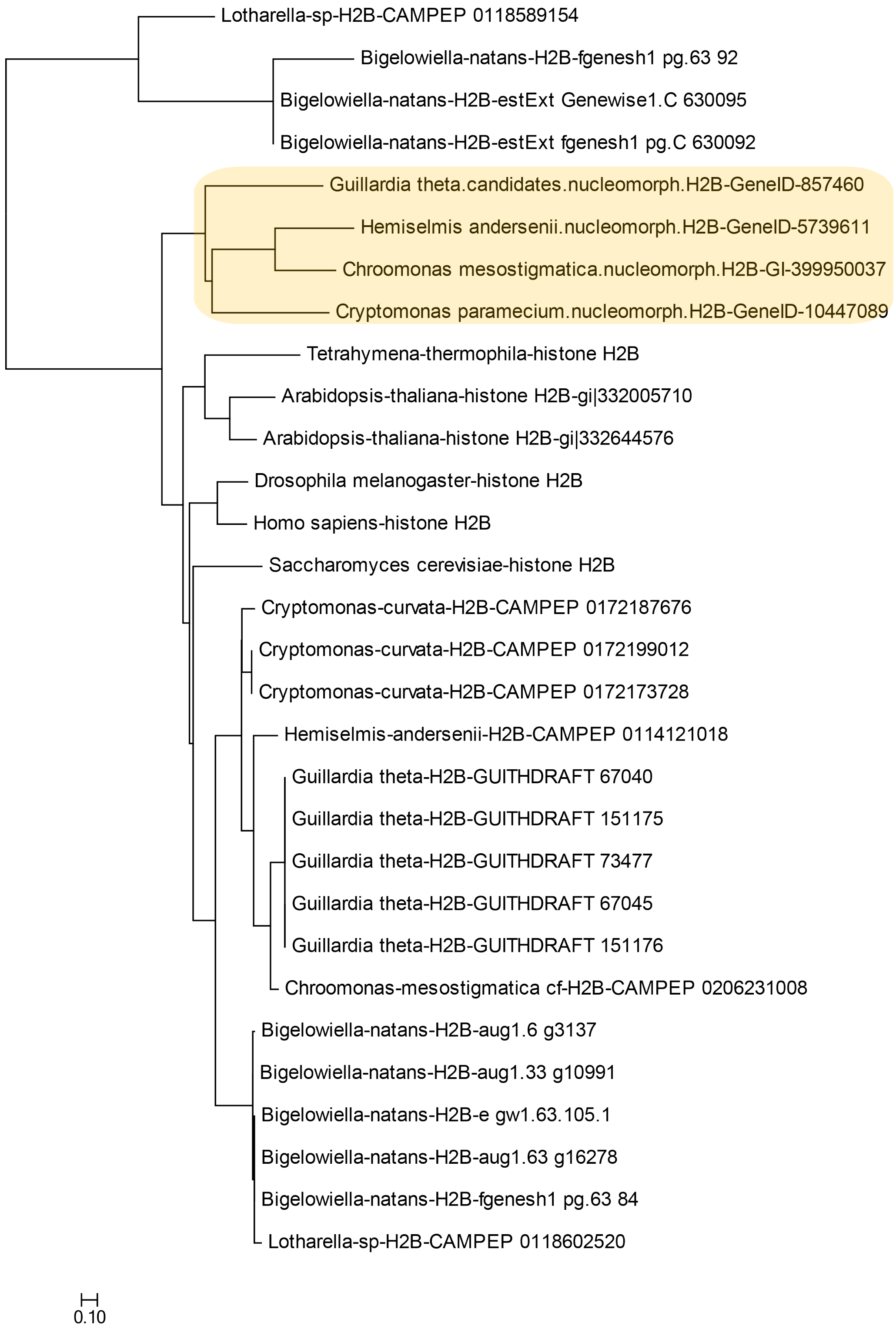

Supplementary Figure 2: Maximum likelihood phylogenetic tree of all nucleus- and nucleomorph-coded H2B protein variants in cryptophytes and chlorarachniophytes. The tree was generated using MUSCLE (Edgar 2004) multiple sequence alignments and RAxML (Stamatakis 2014; version 8.0.26) under the LG+G model and with 100 bootstrap replicates, and visualized using MEGA (Tamura et al. 2013). H2B sequences were obtained from nucleomorph genome assemblies, from available genome assemblies for *Bigelowiella natans* and *Guillardia theta* and from transcriptome assemblies for *Lotharella* sp., *Hemiselmis andersenii*, *Cryptomonas paramecium*, and *Chroomonas mesostigmatica* provided by the Marine Microbial Eukaryote Transcriptome Sequencing Project (MMETSP) (Keeling et al. 2014) (after filtering out incompletely assembled proteins). Canonical H2A sequences from several other eukaryotes were also included.

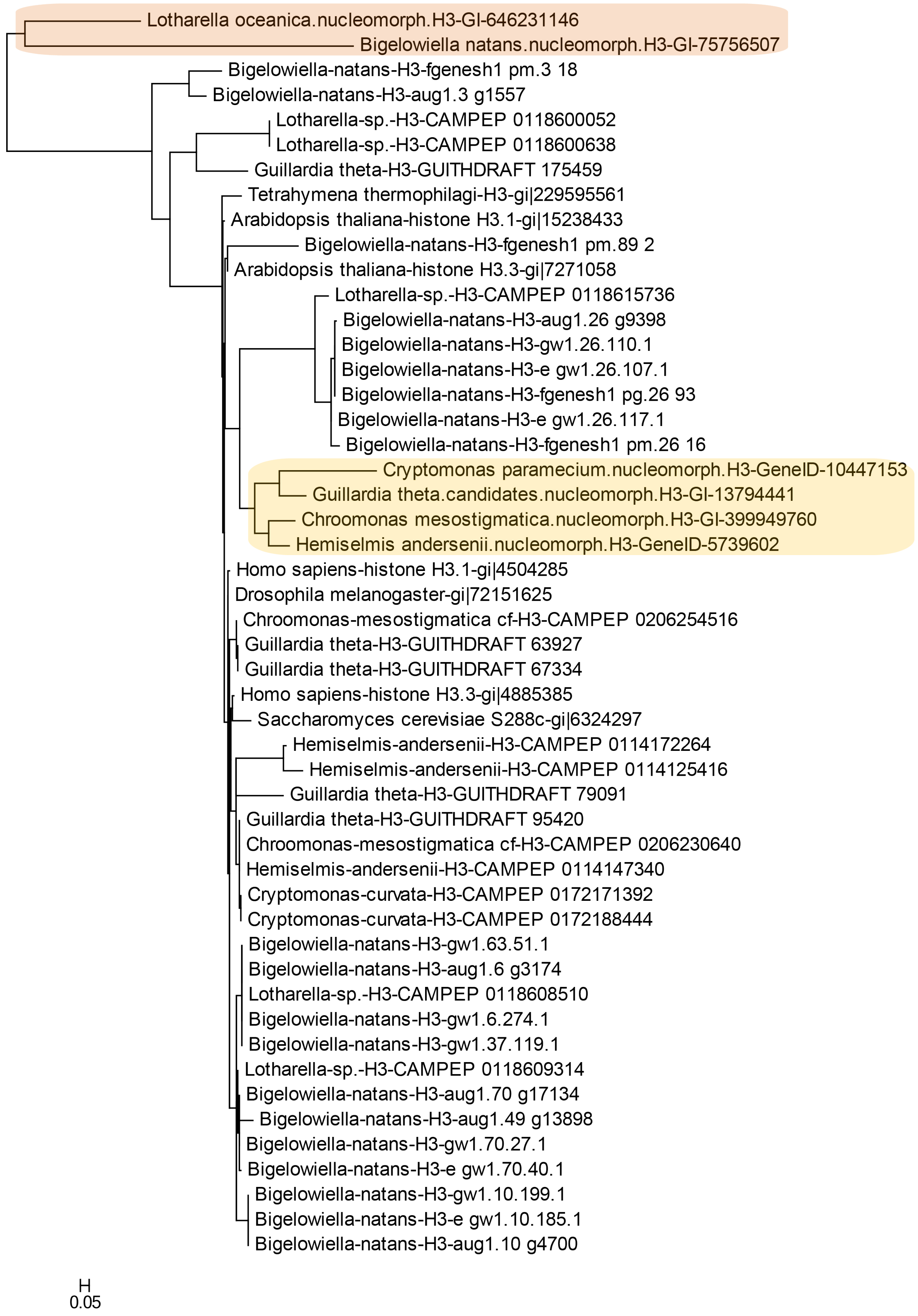

Lotharella oceanica.nucleomorph.H3-GI-646231146
Bigelowiella natans.nucleomorph.H3-GI-75756507
Bigelowiella-natans-H3-fgenesh1 pm.3 18
Bigelowiella-natans-H3-aug1.3 g1557
Lotharella-sp.-H3-CAMPEP 0118600052
Lotharella-sp.-H3-CAMPEP 0118600638
Guillardia theta-H3-GUITHDRAFT 175459
Tetrahymena thermophilagi-H3-gi|229595561
Arabidopsis thaliana-histone H3.1-gi|15238433
Bigelowiella-natans-H3-fgenesh1 pm.89 2
Arabidopsis thaliana-histone H3.3-gi|7271058
Lotharella-sp.-H3-CAMPEP 0118615736
Bigelowiella-natans-H3-aug1.26 g9398
Bigelowiella-natans-H3-gw1.26.110.1
Bigelowiella-natans-H3-e gw1.26.107.1
Bigelowiella-natans-H3-fgenesh1 pg.26 93
Bigelowiella-natans-H3-e gw1.26.117.1
Bigelowiella-natans-H3-fgenesh1 pm.26 16
Cryptomonas paramecium.nucleomorph.H3-GeneID-10447153
Guillardia theta.candidates.nucleomorph.H3-GI-13794441
Chroomonas mesostigmatica.nucleomorph.H3-GI-399949760
Hemiselmis andersenii.nucleomorph.H3-GeneID-5739602
Homo sapiens-histone H3.1-gi|4504285
Drosophila melanogaster-gi|72151625
Chroomonas-mesostigmatica cf-H3-CAMPEP 0206254516
Guillardia theta-H3-GUITHDRAFT 63927
Guillardia theta-H3-GUITHDRAFT 67334
Homo sapiens-histone H3.3-gi|4885385
Saccharomyces cerevisiae S288c-gi|6324297
Hemiselmis-andersenii-H3-CAMPEP 0114172264
Hemiselmis-andersenii-H3-CAMPEP 0114125416
Guillardia theta-H3-GUITHDRAFT 79091
Guillardia theta-H3-GUITHDRAFT 95420
Chroomonas-mesostigmatica cf-H3-CAMPEP 0206230640
Hemiselmis-andersenii-H3-CAMPEP 0114147340
Cryptomonas-curvata-H3-CAMPEP 0172171392
Cryptomonas-curvata-H3-CAMPEP 0172188444
Bigelowiella-natans-H3-gw1.63.51.1
Bigelowiella-natans-H3-aug1.6 g3174
Lotharella-sp.-H3-CAMPEP 0118608510
Bigelowiella-natans-H3-gw1.6.274.1
Bigelowiella-natans-H3-gw1.37.119.1
Lotharella-sp.-H3-CAMPEP 0118609314
Bigelowiella-natans-H3-aug1.70 g17134
Bigelowiella-natans-H3-aug1.49 g13898
Bigelowiella-natans-H3-gw1.70.27.1
Bigelowiella-natans-H3-e gw1.70.40.1
Bigelowiella-natans-H3-gw1.10.199.1
Bigelowiella-natans-H3-e gw1.10.185.1
Bigelowiella-natans-H3-aug1.10 g4700
0.05

Supplementary Figure 3 *(preceding page)*: Maximum likelihood phylogenetic tree of all nucleus- and nucleomorph-coded H3 protein variants in cryptophytes and chlorarachniophytes. The tree was generated using MUSCLE (Edgar 2004) multiple sequence alignments and RAxML (Stamatakis 2014; version 8.0.26) under the LG+G model and with 100 bootstrap replicates, and visualized using MEGA (Tamura et al. 2013). H3 sequences were obtained from nucleomorph genome assemblies, from available genome assemblies for *Bigelowiella natans* and *Guillardia theta* and from transcriptome assemblies for *Lotharella* sp., *Hemiselmis andersenii*, *Cryptomonas paramecium*, and *Chroomonas mesostigmatica* provided by the Marine Microbial Eukaryote Transcriptome Sequencing Project (MMETSP) (Keeling et al. 2014) (after filtering out incompletely assembled proteins). Canonical H2A sequences from several other eukaryotes were also included.

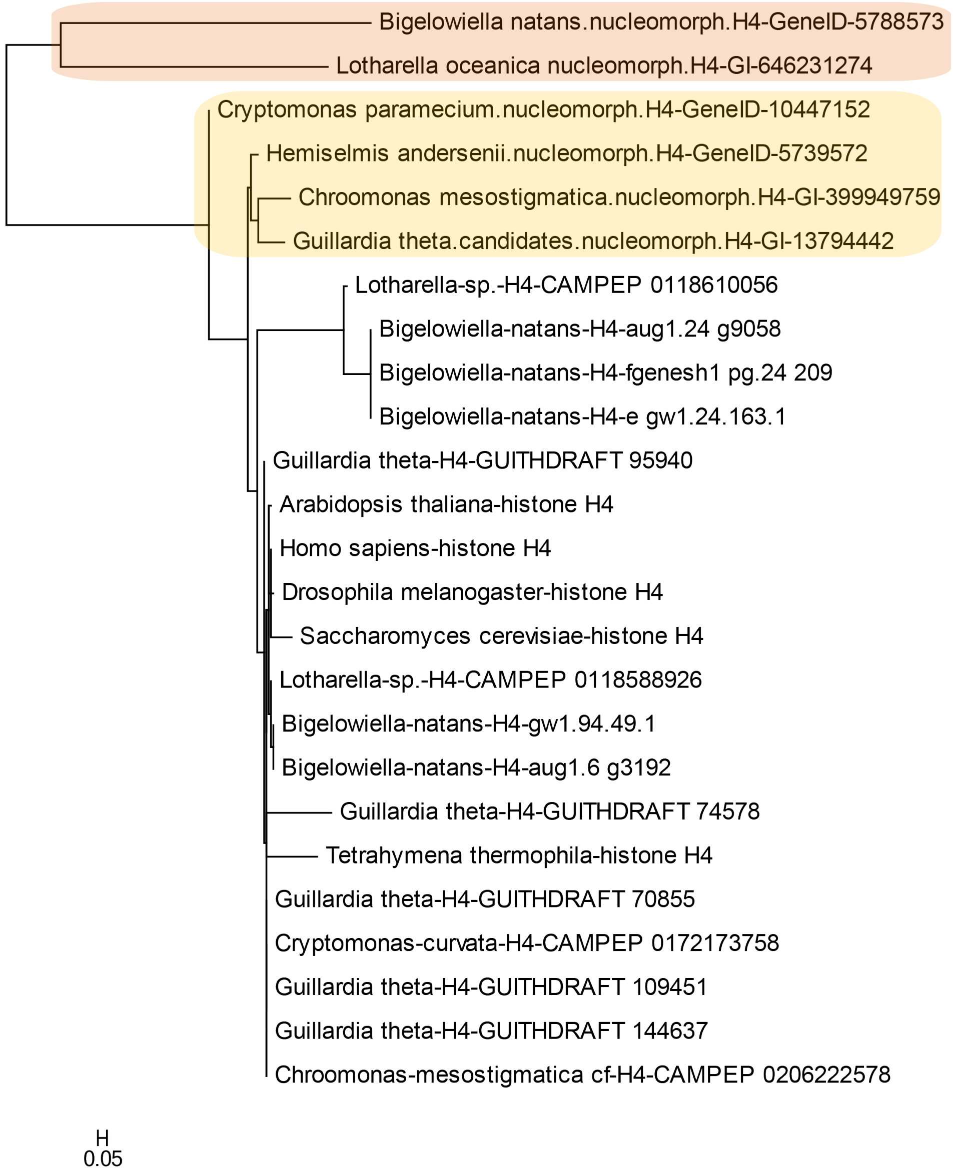

Supplementary Figure 4: Maximum likelihood phylogenetic tree of all nucleus- and nucleomorph-coded H4 protein variants in cryptophytes and chlorarachniophytes. The tree was generated using MUSCLE (Edgar 2004) multiple sequence alignments and RAxML (Stamatakis 2014; version 8.0.26) under the LG+G model and with 100 bootstrap replicates, and visualized using MEGA (Tamura et al. 2013). H4 sequences were obtained from nucleomorph genome assemblies, from available genome assemblies for *Bigelowiella natans* and *Guillardia theta* and from transcriptome assemblies for *Lotharella* sp., *Hemiselmis andersenii*, *Cryptomonas paramecium*, and *Chroomonas mesostigmatica* provided by the Marine Microbial Eukaryote Transcriptome Sequencing Project (MMETSP) (Keeling et al. 2014) (after filtering out incompletely assembled proteins). Canonical H2A sequences from several other eukaryotes were also included.

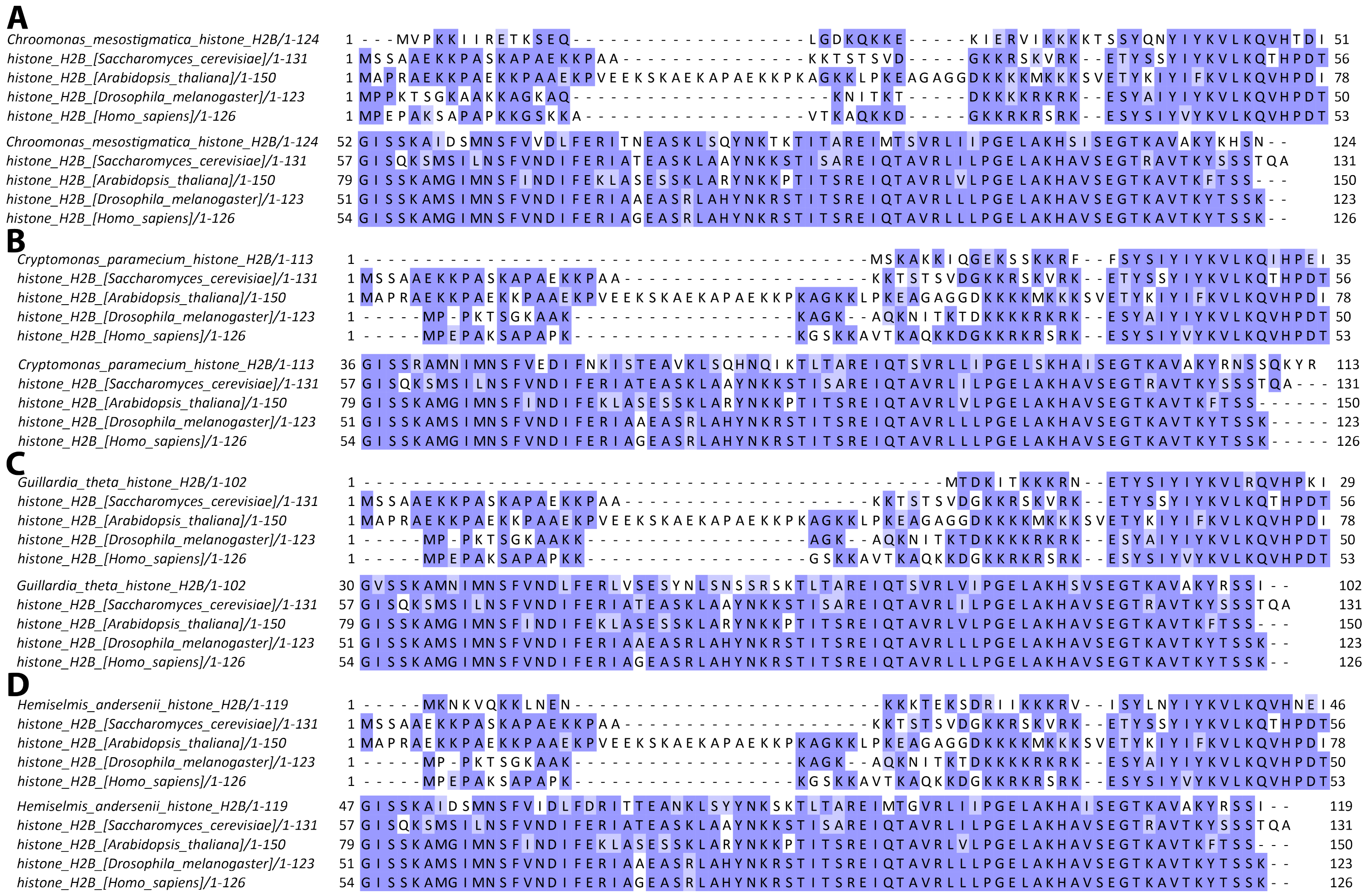

Supplementary Figure 5: Alignment of cryptophyte nucleomorph H2B histones and the nuclear H2B histones from several representative eukaryotes. (A) *Chroomonas mesostigmatica* histone H2B; (B) *Cryptomonas paramecium* histone H2B; (C) *Guillardia theta* histone H2B; (D) *Hemiselmis andersenii* histone H2B.

H2B	*Chroomonas mesostigmatica*			*Cryptomonas paramecium*			*Guillardia theta*			*Hemiselmis andersenii*		
radius:	0	1	2	0	1	2	0	1	2	0	1	2
K 5	0	0	0	0	0	0	0	0	0	0	0	0
K 12	0	0	0	0	0	0	0	0	0	0	0	0
K 15	0	0	0	0	0	0	0	0	0	0	0	0
K 20	0	0	0	1	0	0	0	0	0	1	0	0
K 116	1	1	1	1	1	1	1	1	1	1	1	1
K 120	1	0	0	1	0	0	1	0	0	1	0	0

Supplementary Figure 6: Conservation of key posttranscriptionally modified residues in the cryptophyte nucleomorph histone H2B. The radius r refers to the size of the context considered when scoring conservation. When $r = 1$, only the residue itself is considered; when $r = 1$, a perfect match to the three-amino acid peptide also including the flanking residues on each side is required; when $r = 2$, the five-amino acid peptide also including the two flanking residues on each side is considered. A score of 1 means conservation according to these criteria, while a score of 0 means lack of conservation. Conservation was scored against the *Homo sapiens* histone H2B sequence.

```
RPB1_Saccharomyces_cerevisiae/1-1733     1 --MVGQQYSSAPLRTVKEVQFGLFSPEEVRAISVAKIRFPETMDETQTRAKIGGLNDPRLGSIDRNLKCQTCQEGMNECPGHFGHIDLAKPVFHVGFIAKIKKVCECVCMHC 110
Bigelowiella_natans/1-1510               1 MDFKRYTKSKAKIKKPIAIVYGILNSDEIKRYSIHCFNNKETLDI--SR-TLENLDIPKMGTIENTSICPLDSNTGKDCPGYFGHIELFLPVFNCNYLGLILKILKSVNYYT 109
Lotharella_oceanica/1-1502               1 MDSHRFSCSKAPFSNIGSIQFGIWNPDEIKKYSIKNITFSQIGNK--LRPSIDELNSEKFGVLEKNQLCSVDESNLINCPGYFGHVELTKPVYNINYLSIVKKILQSINFYT 110
Guillardia_theta/1-1473                  1 --------MVREEKLIKLIQFGILSPDEILKLSVVQVDSDMTFEH--NLPKLGGLLDQRMGTVDKEFFCTTDMGSFLESPGYFGHISLNRPVFHEGFMDYVLRILRCIDHTT 102
Cryptomonas_paramecium/1-1452            1 ---------MIEEKKINSIQFSILSPDEIKKYSVVNVETDLISEE--NLPKKGGLMDTRLGVIEQGYYCETDMASHIECPGHFGHINLQKPIFHEGFIEFVLLIIRCIDHMS 101
Chroomonas_mesostigmatica/1-1473         1 -----MYYDIVEEKKIDYIQFSILSPEEIRIYSVAKIENDFTYQN--SIPKMGGLMDPRLGAVDQNICCTTDMNEFIECPGYFGHLELAKPVFHESFMDFVLMILRCVDHVT 105
Hemiselmis_andersenii/1-1464             1 ----MMVNDQIEEKKVKYIQFSILSPQEIRNYSVACIESDLSFEN--NSPKTGGLMDPRLGAVDKDIYCTSDMNSFVDCPGYFGHLELAKPVFHESFMNLVLLILRCIDHST 106

RPB1_Saccharomyces_cerevisiae/1-1733   111 GKLLLDEHNELMRQALAIKDSKKRFAAIWTLCKTKMV----CETDVPSEDDPTQLVSRGGCGNTQPTI-RKDGLKLVGSWKKDRATGDADEPEL---RVLSTEEILNIFKHI 214
Bigelowiella_natans/1-1510             110 SALLIEKTRTNIQELLK-HNISDRISIISK-----------EADRVKTDLLSGDFL---------PEYESLKGIQIWMRFPLCVKQGKF-NPNILVKRKLFPEQAYSILKNI 199
Lotharella_oceanica/1-1502             111 SEILIDYNTVEMRSVLKEKNFSKRINLFVK-----------YSNKIKNDKFSGEPL---------PTY-KIDGTTIFIEFPTMTASRTF-DYKLVQRSELKPEKAYEILKKI 200
Guillardia_theta/1-1473                103 SKLIINENSQKIKNVLKIKNLSQRLNIISKLCLTACNLKKNKFNNFLKDEIIEKRIS------IQPKY-SRDGWCIVAKFDSRITS----EPT----RLVSAERIHEIFVKI 199
Cryptomonas_paramecium/1-1452          102 SKLIFNQNSQRVKNVMKIKNPYQRLNINIAKLCVSSFGYR--VFNKNSSDTFRQTVI--------QPKY-YRDGWCIVAKFDPKITS----EPT----RILSAERIYEIFKRI 194
Chroomonas_mesostigmatica/1-1473       106 SRLIFNCDSPKVKNILKIRNPRQRLSMLSKLGSTSTGLK--SEKNTSSDDFFEENIT------YQPKY-SRDGWCIIAKFNQSITS----EPT----RILGAERIHEIFEKI 200
Hemiselmis_andersenii/1-1464           107 SKLILNSKSQKVKNILKVRNPKQRLSILSKLCSSLNSSK--TEKNVSNDDFFEENLT------FQPKY-SRDGWCIIAKFDKFITS----EPT----RIMSAERIHEIFKNI 201

RPB1_Saccharomyces_cerevisiae/1-1733   215 SVKDFTSLGFNEVFSRPEWMILTCLPVPPPPVRPSISFNESQRGEDDLTFKLADILKANISLETLEHNGAPHHAIEEAESLLQFHVATYMDNDIAGQPQALQKSGRPVKSIR 326
Bigelowiella_natans/1-1510             200 SKFDCNILGFDFKNSRPNWLILYNLPIPPINIRPYSKKNLFIKYEDDLTYKLHDIIKANDRLQRSKNSRKSSELLENYRELLSYHIFTYFDNSSTKVKLSTHVSGKPIKSIS 311
Lotharella_oceanica/1-1502             201 SKKNCILLGFDYYNIRPNWFILYNLPVPPIYVRPYCKKNFIQRSEDDLTYKLCDIIKVNERLLKVIIAKKPISLVNHYRELLAYHVSTYFDNSSLKLPISTQTSGRPIKGIV 312
Guillardia_theta/1-1473                200 TDEACEKLGLNPKLSRPDWMIITVLPVPPPTIRPSVKFETLLRAEDDLTYKLGDIIRTNKILRKAIFSCLPSQLINEQINLLQYHIGSYMNNSVPSLAKSLQKSGRPIKSIK 311
Cryptomonas_paramecium/1-1452          195 SDQDCNKIGLNPKLARPDWMILTVLPVPPPSIRPSIIFDSTSRAEDDLTYKLSDIIRCNKSLSHLIISYAPLQLINEQINLLQYHVGSYMDNTTPNLAKSIQKTGRPIKSIR 306
Chroomonas_mesostigmatica/1-1473       201 SDLDSKKIGLNPSVSKPDWMILTVLPIPPPTIRPSVMFDMTSRAEDDLTYKLGDIVRTNKSLFQLTIACAPVQIINEHLNLLQYHIGSYMNNKVPNIPKSIQKSGRPIKSLA 312
Hemiselmis_andersenii/1-1464           202 VDLDCKKIGLNPKLCRPDWMILTVLPVPPPTIRPSVMFDMTSRAEDDLTYKLGDIIRTNKSLSQLLISCAPAQVINEQFNLLQYHIGSYMNNKVPNIAKSIQKSGRPIKSLA 313

RPB1_Saccharomyces_cerevisiae/1-1733   327 ARLKGKEGRIRGNLMGKRVDFSARTVISGDPNLELDQVGVPKSIAKTLTYPEVVTPYNIDRLTQLVRNGPNEHPGAKYVIRDSGDRIDLRYSKRAGDIQLQYGWKVERHIMD 438
Bigelowiella_natans/1-1510             312 QRLKGKFGRFRRNLVGKRVDFTGRTVITGDSYIHLEELGIPMLIAKKLTLPEVVNKFNIDRLSYII-DSTGNELKANYIIKGYNKVIDLRYKSSNSIILLKYGFIIERHIQS 422
Lotharella_oceanica/1-1502             313 QRLNGKFGRLRRNLMGKRVDFTGRTVITGDSYINLDELGIPKIISKKLTIPTIVHKFNIERMSTLV-DSISKDLKANSILKGYNKKIDLRYKSATSIILLKYGFIIEKHLED 423
Guillardia_theta/1-1473                312 QRLQGKEGRLRGNLMGKRVDFSARTVITPDPNIKLDELGVPLSIAMNLTFPEIVTSFNIEYMKFLLYNGPYKHPGAKYLIRNDNNRIDLRYVKYLSNINLENGYCVERHLQN 423
Cryptomonas_paramecium/1-1452          307 QRLQGKEGRIRGNLMGKRVDFSARTVITPDPNILLEEIGIPWNVALNLTYPEIVTNFNYQYMKQLVQNGPTRYPGAKYVIKNDGTRLDLRYVKKLSNVYLEIGYQVERHLRD 418
Chroomonas_mesostigmatica/1-1473       313 ERLQGKEGRVRGNLMGKRVDFSARTVITPDPNIPLDGIGVPWSIALNLTYPEIVTSFNFEKLKKLVLNGPIHHPGARYVIKNDGSRLDLRYVKKLDEIPLEIGCKVERHLQD 424
Hemiselmis_andersenii/1-1464           314 QRLQGKEGRVRGNLMGKRVDFSARTVITPDPNILLDEIGIPWSIALNLTYPEIVSEFNMKKMKKIVQNGPIHHPGAKYIIRNDGSRIDLRFVKNLSEIKLEIGNQIERHLQD 425

RPB1_Saccharomyces_cerevisiae/1-1733   439 NDPVLFNRQPSLHKMSMMAHRVKVIPYSTFRLNLSVTSPYNADFDGDEMNLHVPQSEETRAELSQLCAVPLQIVSPQSNKPCMGIVQDTLCGIRKLTLRDTFIELDQVLNM- 549
Bigelowiella_natans/1-1510             423 GDIVILNRQPSLHKMSMMGHRVKIFPKLTFRLNLSVTTPYNADFDGDEMNIHIPQSELTRSESTFIMNVINNIMSPQANKPVIGLVQDTLSTCYTLSSRDSFLNSLEKDYL- 533
Lotharella_oceanica/1-1502             424 GDVLILNRQPSLHKMSMMGHKIKILPYLTFRLNLSVTTPYNADFDGDEMNIHVPQTILTKIESMMIMTVVENIMSPQANKPVIGIVQDSMISSYDLFRKDNFLTKKSLNIVS 535
Guillardia_theta/1-1473                424 GDLVLFNRQPSLHKMSMMGHIVKVMKFSTFRLNLSATSPYNADFDGDEMNLHFPQNVESKSELKNLLMVSNCIVSPQGNKPIMGIVQDSLLGSMLITEKGNFLSKYEIMAY- 534
Cryptomonas_paramecium/1-1452          419 GDVILFNRQPSLHKMSMMSHIVKIMDYSTFRMNLSATTPYNADFDGDEMNLHLPQTLESTSELLNLLTVSKCILSQQSNKPVIGIVQDSLIGAFLLTQKDVFMDMCTLNML- 529
Chroomonas_mesostigmatica/1-1473       425 GDVVLFNRQPSLHKMSMMGHVVKIMHYSTFRMNLSATSPYNADFDGDEMNLHLPQTIEAKAELLNLLMLPNCIISQQGNKPVMGIVQDSLVGGFLLSHRDSFLNRSQFMLL- 535
Hemiselmis_andersenii/1-1464           426 GDLVLFNRQPSLHKMSMMGHIVKIMHFSTFRMNLSATSPYNADFDGDEMNLHLPQTLEGKSELLNLLMLPNCIISQQGNKPVIGIVQDSLLGSFLLSQRNIFLDYSKFMLL- 536

RPB1_Saccharomyces_cerevisiae/1-1733   550 -LYWVPDWDGVIPTPAIIKPKPLWSGKQILSVAIPNGIHLQR----FDEGTTLLSPKDNGMLIIDGQIIFGVVEKKTVGSSNGGLIHVVTREKGPQVCAKLFGNIQKVVNF 656
Bigelowiella_natans/1-1510             534 ----KNNYKNFSTQPSIIKPLKKWTGKQLLSSFLPHLTFLHRESRKTFHFEDSNNYNDSSMYIQDGKIMMGLFNKKTIGASYNSIIQNIWLEYDHFSCRNFIDLCQTTARHT 641
Lotharella_oceanica/1-1502             536 TFDKLRGYN--KKHPSIIKPKMLWSGKQSINYMTTKTSFIIKNIKENDVLWKEINPEDSLIYMFKGNLMMGRLTKKNIGPSYNSIIQNLWTDYSKNESKKVINNLQYSTKQ 645
Guillardia_theta/1-1473                535 -LNLIEGYSLNIPLPAIYKPRILWTGKQLISTIIPCINLSRTCISHPEHEKAYVTSGDTRVIIFQGQLLSGMIDKRTIGATSGGLIHVIWKDIGPSACSYFISNFQIIVNN 645
Cryptomonas_paramecium/1-1452          530 -LIHIDGCN-SIPFPTFIKPNILWTGKQVFSLILPSVNLTRNCINYSDTETNF--SQDNKVIILNGQLICGVIDKRSAGSSSGGIIHVCWKEYGPEKTAFFISQFQWMINH 637
Chroomonas_mesostigmatica/1-1473       536 -LMQLQEWNGSIPFPTILKPRILWTGKQIFSVFLPKINFLRKSLGHSEHEKSNISPRDTRVLILGGHLISGIIDKRSVGASGGSLIHICWKEFGPKKASLFVSQFQLIVNT 646
Hemiselmis_andersenii/1-1464           537 -LMQLQEWDGSIPIPSIIKPQTLWTGKQVFSLFLPKINFLRKCLSHSENEKTNISPKDTRVMILGGQLISGMIDKRSVGASGGSIIHICWKEFGPKKTSNFISQFQTIVNS 647

RPB1_Saccharomyces_cerevisiae/1-1733   657 LLHNGFSTGIGDTIADGPTMREITETIAEAKKKVLDVTKEAQANLLTAKHGMTLRESFEDNVVRF--LNEARDKAGRLAEVNLK----DLNNVKQMVMAGSKGSFINIAQMS 762
Bigelowiella_natans/1-1510             642 NIKNGFTVSLWDVFLEKKLFNIFY----NIKKFYIHNWIKLMETKKQIKMTQIKNFKF-NTITKINNLSTTKKYFSSLINLFLNLTHNQNNNMNIMLNSGSKGSLINLIQIV 748
Lotharella_oceanica/1-1502             646 IFLKGF  SLADLFLDVSMIEIYCTSIENIEKLWLKELRSTFLYDLKFFFGKNKQQYL---KVKT--INEEIKKFDKILSYYLNKGNLNANSLINMMKSGSKGSEINLMQII 752
Guillardia_theta/1-1473                646 LQLNGFSVGIGDCIPTKMVKKDVSLIIKTAKLFVKKSIKRLLSKKFNGFFNKDSNYNLENHLNKV--LNNARDMAGSSAQKCLT----SYNNLKKMIFSGSKGSLINISQIV 751
Cryptomonas_paramecium/1-1452          638 LLLCGFSVGVEDTIANKIINLKVVSIIKKAKNEVRYILRKKVTSA-EIYPDKNSDEIFECQINKI--LNTARDQAGSIVQKLLS----DKNNIKKMVISGSKGNFINISQII 742
Chroomonas_mesostigmatica/1-1473       647 LQSKGFSVGIGDTISDNKIMKDITKTIKKAKKEARQITQKNTNKKKEISIGKILNDNFENHINVI--LNSARDKAGSIAQRCLL----DSNNIKRMVMCGSKGSFINISQII 752
Hemiselmis_andersenii/1-1464           648 LLFEGFSVGIGDTIADKKTMNNIIKTIKSAKIEARQVTLLNINKSEENFLGKILKDDFENHINKI--LNSARDKAGSNAQKSLS----NANNIKRMVDCGSKGSFINISQII 753

RPB1_Saccharomyces_cerevisiae/1-1733   763 ACVGQQSVEGKRIAFGFVDRTLPHFSKDDYSPESKGFVENSYLRGLTPQEFFFHAMGGREGLIDTAVKTAETGYIQRRLVKALEDIMVHYDNTTRNSLGNVIQFIYGEDGMD 874
Bigelowiella_natans/1-1510             749 GLLGKQSSIKINNMNHFHGRLFCHNIIGHIPIESTGFILSNFFYGLNPHEYFCHAISGREGIIDTSIKTAETGYIQRKFSKSMENLICYYDGTVRNSSQSIIQFLYGEDGLD 860
Lotharella_oceanica/1-1502             753 GLLGQQSLVLSEFSRDYKARLFSHYTFFEIPLEIKGYIKNNFLKGLSPYEFFFHSIAGREGIIDTSIKTAETGYIQRKLSKSMENVISYYDGSVRNSKNFIIQFVYGEDGFD 864
Guillardia_theta/1-1473                752 ACVGQQNVEGKRIPFGFRKRSLPHYHYKNFEPETRGFVVNSYIEGLFPDEFFFHSMGGREGLIDTAIKTSETGYIQRRLSKSMEDIVVEYDFSVRNNQQEIISFLYGEDCFD 863
Cryptomonas_paramecium/1-1452          743 ACVGQQNVNGKRIDSAFGSRCLPHFLFDDNGPETKGFVQNSYSSGLNPDEFFFHAMGGREGLIDTAIKTSETGYIQRRLSKIMEDIVVEYDLTVRNAQKEILEFFYGEDSID 854
Chroomonas_mesostigmatica/1-1473       753 ACVGQQNIEGKRIPLCFDNRCLPHFSKQNNGPETRGFVQNSYISGLSPEEFFFHAMGGREGLIDTAIKTSETGYIQRRLSKAMENISVEYDLTVRNSEGGIIEFFYGEDGMD 864
Hemiselmis_andersenii/1-1464           754 ACVGQQNVEGKRIPMGFNKRSLPHFSKDNYGPETRGFVQNSYISGLRPDEFFFHSMGGREGLIDTAIKTSETGYIQRRLSKAMENVMVEYDFTVRNCEGNIIEFFYGEDGMD 865

RPB1_Saccharomyces_cerevisiae/1-1733   875 AAHIEKQSLDTIGGSDAAFEKRYRVDL---LNTDHTL---DPSLLESGSEILGDLKLQVLLDEEYKQLVKDRKFLREVFVDG-EANWPLPVNIRRIIQNAQQTFHIDHTKPS 979
Bigelowiella_natans/1-1510             861 PTFLEKITLSKNHKKQTNKNLQSNLKLKTYDTKDIGF----SNNFSHIHFLNTNFKKYYTQDKILNKIIKQFTMLKRLK------TCYIPVNIIRIIAHTKSSLQ------- 955
Lotharella_oceanica/1-1502             865 PTLIEE-------ISCVENNKNFFMKIFLNNKKCFNFNILCNNKINCKNFYKDKNNVLGIDGFFYHNIINIIKRKKNFLIKK---KFYLPLNIEKIIFRSKMFFT------- 959
Guillardia_theta/1-1473                864 AVYLENQKIFLGKMSDIEMNMVYKVTQ---SSPCFGLAI-DSSLL----FLSNKSYNSKIANIEYNQLLKDREFLRKLMLDQNDINIPLPVNLDRIVSKASVLFK-NHKNSS 966
Cryptomonas_paramecium/1-1452          855 AVFLENQPLNFANITYEELRISYKMDV---KSPCYGLNT-KGRFLLKLNSKQFFEKLQGQLDFEFLQLTRDRHILQQIFSSGIEINLFLPVNIQRLIMNANNFFR-NIETST 961
Chroomonas_mesostigmatica/1-1473       865 ALNLENQQMNTIKMSNREIEIVFRLNV---DSPCLGLDQ-KGNLYKNLSRIKNRKKNREKIIKEFLQITKDRDILRQMSINEYDFNIPLPVNISRIIINANNIFP-KISKYS 971
Hemiselmis_andersenii/1-1464           866 ALNLENQNFDSTKLSDRELEIVFRFNI---NSPLIGMAY-DGKEVSELKIFKDKNTKKKIVE-EFLQIKKDREILRQMIINNYELTIPLPVNIDRIIKNAEKLFP--QEKNC 970

RPB1_Saccharomyces_cerevisiae/1-1733   980 DLTIKDIVLGVKDLQE--------NLLVLRGKNEIIQNAQRD--AVTLFCCLLRSRLATRRVLQEYRLTKQAFDWVLSNIEAQFLRSVVHPGEMVGVLAAQSIGEPATQMTL 1081
Bigelowiella_natans/1-1510             956 RYGKIQKNCTCPNLLSIYLGKLY-NSLSLSRKTCYFKALFNKI-INPSIVLIIKNIFTVKLI-KSLKINLFQLKWIFKQIENKYNKSLTTPGECVGVIGAQSIGEPTTQMTL 1064
Lotharella_oceanica/1-1502             960 DRALKQTVCD-NNLLALFLNTLI-KVLILRTNGVIFNNFKNNF-INFSLLVYI-IRFIDWGNYKKSNITPSSLLWILHKIEKKILKSLICPGLCLGVIAAQSIGEPTTQMTL 1067
Guillardia_theta/1-1473                967 LINFKETIRSLKFLRNYFLELIN-FKISFNQKKSDNKQLINR--DNFLLAIYIRSSLSLKIIYTKFNLNQHALNWIVNEICLSIRKSLIQPGEMVGTISAQSIGQPATQMTL 1075
Cryptomonas_paramecium/1-1452          962 SLFPLDIINSVKCVEKFLLNTLY-RHKKNCKKNCVCCECLSVFNATLFFRMYLRIIFASKSVIYKHKFTKEKFSWILNEICLYFRKSIISPGEMVGMISSQSIGQPATQMTL 1072
Chroomonas_mesostigmatica/1-1473       972 DLSPTEIIDGVKKIENYCFDSLGPKIDSLCKSFCNCCRCESRLNATLLFCIYLRSMLASKLVLFTYKLSRKSFVWVLNEICIQYRKSIINPGEMVGTISAQSIGQPATQMTL 1083
Hemiselmis_andersenii/1-1464           971 RISPIEIIDGLKKLENYCFDSLGPKKNEECSGFCNCCKCESRLNATLLFRIFLRSKLASKIIFFKYKFSKVAFLWVLNEICTQYRKSIASPGEMVGTISAQSIGQPATQMTL 1082

RPB1_Saccharomyces_cerevisiae/1-1733  1082 NTFHFAGVASKKVTSGVPRLKEILNVAKNMKTPSLTVYLEPGHAADQEQAKLIRSAIEHTTLKSVTIASEIYYDPDPRSTVIPEDEEIIQLHFSLLDEEAEQSFDQQSPW-- 1191
Bigelowiella_natans/1-1510            1065 NTFHYAGIAEKDVTTGVPRFNELINATKKPKTKCSYLSLIHNSKLEIESTDFIISRLEETKLEFFVEKLYLLKVFDSQYTRIDFISSILKNSLESINPSSK--SDQNPRW-- 1172
Lotharella_oceanica/1-1502            1068 NTFHHAGILDKNVTVGVPRFNEIINVSKNIKHTIVNVCLTSQNSLEFESSKFIISRIKYRKFIDILSNIEVLY----LNYIKIDDRINNINFFKMPLMNVFLNYNGKDAFVN 1175
Guillardia_theta/1-1473               1076 NTFHFAGVSAKNVTLGVPRLKEIIDIQKNIKSPSLTIFLHKKCSNDPRKVKKIQESIESVRLNRISNYFEIIYDPDFFFSISKCNRILVKNYMEIPDEDIN--FLQASSW-- 1183
Cryptomonas_paramecium/1-1452         1073 NTFHYAGVSAKNVTLGIPRLKEIINVLKITKTPSLTVYIKKEYNFSYEKVKYLQQFLEFVTLKDMMSSISFFYDPDPFLTICSTDRNVIKQYFEISDQVLE--TTHIGTW-- 1180
Chroomonas_mesostigmatica/1-1473      1084 NTFHYAGVSAKNVTLGVPRLKEIINVSRTAKTPSLTIYFKKNFAFAIGKIKHFQQTLEYLTLKSLMDKSLFIYDPDPYLTVFKKNRFMVENFYEIPEINLD--NTSLGTW-- 1191
Hemiselmis_andersenii/1-1464          1083 NTFHYAGVSAKNVTLGVPRLKEIINVLQTVKTPSLTIYFRKEFSSSLSKIKQFQQTLEFITLKSMIKKKTVFYDPDPYKTILKNDQIILENYFEIPDWASE--ISCIGTW-- 1190

RPB1_Saccharomyces_cerevisiae/1-1733  1192 -LLRLELDRAAMNDKDLTMGQVGERIKQTFKN--------------DLFVIWSEDNDEKLIIRCRVVRPKSLDAETEAEEDHMLK--------------------------- 1261
Bigelowiella_natans/1-1510            1173 -ILKLIISRWRLFEKKISIVKLFKKLSDKLKNYGNIQISFADKKFIEIAIIISNTLFAPYSIKLLDVDPKKKTPHIKERCKLGVR-------------------------KSK 1259
Lotharella_oceanica/1-1502            1176 LIFTLYFDRLKLNNLNLSMVEIIAKITYHYK---------------FKVYIISTSDNIKNIYLQIFYFKSKKINS-----IIKKYK--------------------------K 1242
Guillardia_theta/1-1473               1184 -ILKLKLLTEEFVEKGLFSNYIIETIKKKIKP--------------IPFLIIASDENSGSIEFLVRFIWPEFINNYNSEKIEKLFSRSNANTFFEYIKRANQKVSENLFLEPN 1281
Cryptomonas_paramecium/1-1452         1181 -VMRIILYKNKFIDKNLSLNQIIEKIKRKIKP--------------LVLFTLNSDENNIPNLIRFKIFWPDNKWKKKNMKQHQLYT-------FDYSS-----------LEIQ 1260
Chroomonas_mesostigmatica/1-1473      1192 -VLLLKIQPEEFVHRRLSIEQLVEKIKRKSKP--------------LSLLIINSNENHDCLLMRIKVFWPKFNWSKNLSNESKNFK-------TVFQI-----------FERQ 1271
Hemiselmis_andersenii/1-1464          1191 -TTKIGLEKDEFIDKKLSTIQIVEKMKKKIKP--------------LPLFIVNSDENSQNLFFRLKLIWPNIDWTKNSKKTVKNPN-------FNFFN-----------FERK 1270

RPB1_Saccharomyces_cerevisiae/1-1733  1262 --KIENTMLENI-TLR--GVENIERVVMMKYDRKVPSP--TGEYVKEPEWV-LETDGVNLSEVMTVPGIDPTRIYTNSFIDIMEVLGIEAGRAALYKEVYNVIASDGSYVNY 1365
Bigelowiella_natans/1-1510            1260 FLYILEKFKSEILNIPILGIPDIVHCRVSNKVRTFINN-LNYDLNSLENFILLDCIGINLVKTLTHPWIDSSLTYCNDVNCFYDNFGIEAARLLFIKELKKILEFDNSYINQ 1370
Lotharella_oceanica/1-1502            1243 FENIINFIINDILEIPLFGIAGAREVGISNNIRTISYN-EKNIFKKIENQLFLDLYDCKVLDISMLPWVDVNLIASNNIIDIYEFFGIEASRLLLLLELKKILGQGSTYINM 1353
Guillardia_theta/1-1473               1282 YFKKIKHYFYNY-NLKPYGSQKLTKLYPRRIDIIEPEPNQSGKLKNNFEFV-LETEGVDLRFVMNYNGIDYSRCTTNDLLESLKVLGIEATRKILIQELRNLISFDGSYVNY 1391
Cryptomonas_paramecium/1-1452         1261 YLKKMSFLMMSI-NLKDHGTSNIKKIFIQKTNVNNTVI-KNDQLVGSSEFI-LDTEGIDLKYVLNCNIVDPTRTVSNDILETFKTFGIEAARKILIKEIKNLISFDGSYVNF 1369
Chroomonas_mesostigmatica/1-1473      1272 YLIRLKYLIVNI-NLKIHGTSKIKKALIRKIDFPKFDP-LKGSFRSQKEYV-LDTEGVDLRYILNFPGMDHIRSSSNDIVEIFRILGVEASRRILIQELRNLISFDGSYVNS 1380
Hemiselmis_andersenii/1-1464          1271 YLNRTTYFLSNI-NLKSYGTSKIKKAFIRKSSQIIFES-ISGSIKNNEEYV-LDTEGVDLRYILNYSGIDYTRCSSNDILEIYKILGIEAVRKILIQELRNLISFDGSYVNS 1379

RPB1_Saccharomyces_cerevisiae/1-1733  1366 RHMALLVDVMTTQGGLTSVTRHGFNRSNTGALMRCSFEETVEILFEAGASAELDDCRGVSENVILGQMAPIGTGAFDVMIDEESLVKYMPEQKITEIEDGQDGGVTPYSNES 1477
Bigelowiella_natans/1-1510            1371 HHISCLADYSTNTGRPNAINRYGLKVQSLSTLQECSFEETMKTLLKASVETKVDRFSSITETIIFGKESNFGTGFSNMKKTKFQEVQ------ISKNSFKRSPLIIPSFNQS 1476
Lotharella_oceanica/1-1502            1354 HHILCLTDFMTNLGFLNSINRFGLKYQALSALQQCSFEETAKTFIKASINTKRDELNSITENVILGKLIKLGTGFFNPFVKPQLGNHKIKN--IVKQTLQQTKEINNNSLVI 1463
Guillardia_theta/1-1473               1392 RHLCTLVDLITFRGSLMSINRHGINKCNSGPIAKSTFEETVDIFYESAVFGLNDNLRGVSAQIMTGMVPKIGTGKIDLFVNI------------------------------ 1473
Cryptomonas_paramecium/1-1452         1370 RHIFLLADIMTHGGKLMSITRHGISKIEIGPIAKSSFEETVDIFYQSAVFGIYDNIKGVSANIMVGNLLSVGTGKVDLFVDDF----------------------------- 1452
Chroomonas_mesostigmatica/1-1473      1381 RHLYLLVDIMTHRGKLLSITRHGINKTEIGPIAKCSFEETIDILYQAAAFGISDNLKSISASVLLGQLPLLGTGKIDLFLSEKKLMEKEHTAK------------------- 1473
Hemiselmis_andersenii/1-1464          1380 RHLSLLVDVMTHKGKLMSITRHGINRTDIGPIAKCSFEETIEVLYQAAAFGVCDNLKGVSGSILVGQVSSLGTGKIDLFLCEKKF--------------------------- 1464

RPB1_Saccharomyces_cerevisiae/1-1733  1478 GLVNADLDVKDELMFSPLVDSGSNDAMAGGFTAYGGADYGEATSPFGAYGEAPTSPGFGVSSPGFSPTSPTYSPTSPAYSPTSPSYSPTSPSYSPTSPSYSPTSPSYSPTSP 1589
Bigelowiella_natans/1-1510            1477 KSSYKLVSYYK-----PLIKNKKLQYNPYHQKKFLEWEY------------------------------------------------------------------------- 1510
Lotharella_oceanica/1-1502            1464 FEQYKLYNLFKFKTVNNILSKKNNKINLNSKKYYYDWDY------------------------------------------------------------------------- 1502
Guillardia_theta/1-1473                    ----------------------------------------------------------------------------------------------------------------
Cryptomonas_paramecium/1-1452              ----------------------------------------------------------------------------------------------------------------
Chroomonas_mesostigmatica/1-1473           ----------------------------------------------------------------------------------------------------------------
Hemiselmis_andersenii/1-1464               ----------------------------------------------------------------------------------------------------------------

RPB1_Saccharomyces_cerevisiae/1-1733  1590 SYSPTSPSYSPTSPSYSPTSPSYSPTSPSYSPTSPSYSPTSPSYSPTSPSYSPTSPSYSPTSPAYSPTSPSYSPTSPSYSPTSPSYSPTSPSYSPTSPSYSPTSPNYSPTSP 1701
Bigelowiella_natans/1-1510                 ----------------------------------------------------------------------------------------------------------------
Lotharella_oceanica/1-1502                 ----------------------------------------------------------------------------------------------------------------
Guillardia_theta/1-1473                    ----------------------------------------------------------------------------------------------------------------
Cryptomonas_paramecium/1-1452              ----------------------------------------------------------------------------------------------------------------
Chroomonas_mesostigmatica/1-1473           ----------------------------------------------------------------------------------------------------------------
Hemiselmis_andersenii/1-1464               ----------------------------------------------------------------------------------------------------------------

RPB1_Saccharomyces_cerevisiae/1-1733  1702 SYSPTSPGYSPGSPAYSPKQDEQKHNENENSR 1733
Bigelowiella_natans/1-1510                 --------------------------------
Lotharella_oceanica/1-1502                 --------------------------------
Guillardia_theta/1-1473                    --------------------------------
Cryptomonas_paramecium/1-1452              --------------------------------
Chroomonas_mesostigmatica/1-1473           --------------------------------
Hemiselmis_andersenii/1-1464               --------------------------------
```

Supplementary Figure 7 ***(preceding page)*****: Multiple sequence alignment of RNA Pol II largest subunits (Rpb1) from nucleomorphs and *Saccharomyces cerevisiae* (top row)**. The CTD tail domain and its heptad YSPTSPS repeats are absent from all nucleomorph polymerases.

	HAT						HDAC		HMT			demethylation		reader domains					chromatin remodelling									DNA methylation						histone chaperones			
	Acetyltransferase (GNAT)	Hat1_N	KAT11	MOZ_SAS	NuA4	PCAF_N	Hist_deacetyl	SIR2	SET	DOT1	PRMT5	Jmj	LSD1	Bromodomain	Chromo	PHD	TUDOR	PWWP	SNF2_N	HAND	SLIDE	SnAC	DBINO	SNF2_N + Helicase_C	SWIB	SWIRM	FACT	DNMT	MBD	CXXC	SAD/SRA	Tet/JBP	Methyltransferases	ASF1	CAF-1	Hira	NAP
Bigelowiella natans	**0**	0	0	0	0	0	1	0	0	0	0	0	0	0	0	0	0	0	1	0	0	0	0	1	0	0	0	0	0	0	0	0	0	0	0	1	0
Lotharella oceanica	**0**	0	0	0	0	0	1	0	0	0	0	0	0	0	0	0	0	0	0	0	0	0	0	0	0	0	0	0	0	0	0	0	0	0	0	0	0
Chroomonas mesostigmatica	1	0	0	0	0	0	1	0	2	0	0	0	0	0	0	0	0	0	1	0	0	0	0	1	0	0	0	0	0	1	0	0	0	1	0	1	0
Cryptomonas paramecium	1	0	0	0	0	0	1	0	0	0	0	0	0	0	0	0	0	0	1	0	0	0	0	1	0	0	0	0	0	0	0	0	0	1	0	0	0
Guillardia theta	1	0	0	0	0	0	1	0	2	0	0	0	0	0	0	0	0	0	1	0	0	0	0	1	0	0	0	0	0	0	0	0	0	1	0	1	0
Hemiselmis andersenii	3	0	0	0	0	0	1	0	2	0	0	0	0	1	0	0	0	0	1	0	0	0	0	1	0	0	0	0	0	0	0	0	0	1	0	1	0

Supplementary Figure 8: Presence and absence of epigenetic mark depositing and reading domains as well as histone chaperons, chromatin remodellers, DNA methylation-related proteins and the FACT complex in nucleomorph genomes. Domains were identified using HMMER3.0, the number of proteins containing a significant match to each domain/protein is shown.

Supplementary References

Edgar RC. 2004. MUSCLE: multiple sequence alignment with high accuracy and high throughput. *Nucleic Acids Res* **32**(5):1792–1797.

Keeling PJ, Burki F, Wilcox HM, Allam B, Allen EE, Amaral-Zettler LA, Armbrust EV, Archibald JM, Bharti AK, Bell CJ, Beszteri B, Bidle KD, Cameron CT, Campbell L, Caron DA, Cattolico RA, Collier JL, Coyne K, Davy SK, Deschamps P, Dyhrman ST, Edvardsen B, Gates RD, Gobler CJ, Greenwood SJ, Guida SM, Jacobi JL, Jakobsen KS, James ER, Jenkins B, John U, Johnson MD, Juhl AR, Kamp A, Katz LA, Kiene R, Kudryavtsev A, Leander BS, Lin S, Lovejoy C, Lynn D, Marchetti A, McManus G, Nedelcu AM, Menden-Deuer S, Miceli C, Mock T, Montresor M, Moran MA, Murray S, Nadathur G, Nagai S, Ngam PB, Palenik B, Pawlowski J, Petroni G, Piganeau G, Posewitz MC, Rengefors K, Romano G, Rumpho ME, Rynearson T, Schilling KB, Schroeder DC, Simpson AG, Slamovits CH, Smith DR, Smith GJ, Smith SR, Sosik HM, Stief P, Theriot E, Twary SN, Umale PE, Vaulot D, Wawrik B, Wheeler GL, Wilson WH, Xu Y, Zingone A, Worden AZ. 2014. The Marine Microbial Eukaryote Transcriptome Sequencing Project (MMETSP): illuminating the functional diversity of eukaryotic life in the oceans through transcriptome sequencing. *PLoS Biol* **12**(6):e1001889.

Stamatakis A. 2014. RAxML version 8: a tool for phylogenetic analysis and post-analysis of large phylogenies. *Bioinformatics* **30**(9):1312–1313.

Tamura K, Stecher G, Peterson D, Filipski A, Kumar S. 2013. MEGA6: Molecular Evolutionary Genetics Analysis Version 6.0. *Mol Biol Evol* **30**(12):2725–2729.
